# Management of Cesarean Scar Ectopic Pregnancies: A Retrospective Study and Literature Review

**DOI:** 10.7759/cureus.74515

**Published:** 2024-11-26

**Authors:** Reema Bhatt, Anusmita Saha

**Affiliations:** 1 Department of Fetal Medicine, Amrita Institute of Medical Sciences and Research Centre, Faridabad, IND

**Keywords:** cesarean scar pregnancy, ectopic pregnancy, intracardiac kcl, intrasac methotrexate, minimally invasive technique

## Abstract

Cesarean scar ectopic pregnancy (CSEP) is localized over the scar of a previous cesarean section. CSEP is a challenging entity, both in terms of diagnosis and management. The clinical presentation of CSEP may vary from asymptomatic patients with positive urine pregnancy tests to acute presentations such as pelvic pain, bleeding per vaginum, uterine rupture, and hemodynamic instability. Cesarean scar ectopic pregnancy is primarily diagnosed by transvaginal ultrasound. We present a series of six cases of CSEPs, their diagnostic approaches, and outcomes. Out of our six cases, four patients underwent intracardiac injection of potassium chloride (KCl) followed by methotrexate instillation into the gestational sac. This led to the successful resolution of cardiac activity and the collapse of the gestational sac. Two patients underwent curettage under ultrasound guidance. All of the patients recovered successfully without any major surgery. The key to diagnosis is the high degree of suspicion of CSEP in cases of previous cesarean deliveries, even in the absence of any symptoms.

## Introduction

Cesarean scar ectopic pregnancy (CSEP) is an often encountered clinical entity, especially in tertiary referral centers. Following a cesarean section, there may be impaired wound healing, leading to a myometrial defect and scar formation. The blastocyst of subsequent pregnancy implants into the myometrial scar, thus leading to CSEP [1]. The cesarean scar ectopic pregnancy is localized over the scar of a previous C-section and is completely surrounded by myometrium and fibrotic tissue [2].

CSEP can be divided into two types, Type 1 (endogenic): in this type, the gestational sac grows inwards toward the cervical isthmus space. The clinical presentation of this type may be less severe because of its ability to grow into the uterine cavity, and Type 2 (exogenic): the gestational sac grows outward toward the bladder and abdominal wall [3,4].

With the increase in the availability of ultrasonography and Doppler studies, the detection of cesarean scar pregnancies has improved. Despite that, the acute clinical presentation and diagnostic dilemma still pose a challenge in the management of this clinical entity. In this study, we describe six cases of cesarean scar ectopic pregnancies, along with their treatment approaches and outcomes. Written informed consent was obtained from each of the study participants. 

## Materials and methods

Study design

This study was a retrospective analysis of women diagnosed with cesarean scar ectopic pregnancy between June 2022 and June 2023 at our academic referral center. Demographic data, including age and number of previous cesarean deliveries, along with treatment modalities and outcomes, were collected and analyzed retrospectively. Institutional Ethics Committee, Amrita Hospital, Faridabad, Haryana, issued approval AIMS-IEC-BAS-08-24-001 for this study.

Data collection

The ultrasound images and pregnancy outcomes of all six cases of CSEP were extracted from the medical record software of the Department of Fetal Medicine. The following data of all diagnosed cases of CSEP has been collected: (1) age, (2) number of previous cesarean sections, (3) gestational age at diagnosis, (4) presence or absence of fetal cardiac activity,(5) treatment modality opted,(6) need for any other intervention/secondary treatment, (7) time to reach negative beta-human chorionic gonadotropin (BHCG).

The inclusion criteria were: (1) history of previous cesarean section, (2) cesarean scar ectopic pregnancies diagnosed by ultrasound, and (3) available hospital records of follow-up. The diagnostic criteria for CSEP include (1) the presence of a gestational sac embedded in a previous cesarean scar at the level of opening of the cervix into the body of the uterus (internal os), (2) empty uterine and endocervical cavity, (3) increased vascularity on Doppler, (4) thin myometrium overlying the gestational sac and bladder, and (5) negative "sliding organ sign" [5]. The exclusion criteria include (1) heterotopic pregnancy, (2) CSEP with insufficient diagnostic criteria, (3) patients not willing to participate in the study, and (4) lack of study records.

Statistical analysis

Data has been coded and recorded in MS Excel spreadsheet program. SPSS software, v23 (IBM Corp., Armonk, NY) has been used for data analysis. Descriptive statistics have been elaborated in the form of means/standard deviations and medians/inter-quartile ranges (IQRs) for continuous variables, and frequencies and percentages for categorical variables. Values of p < 0.05 were considered statistically significant.

## Results

**Figure 1:**
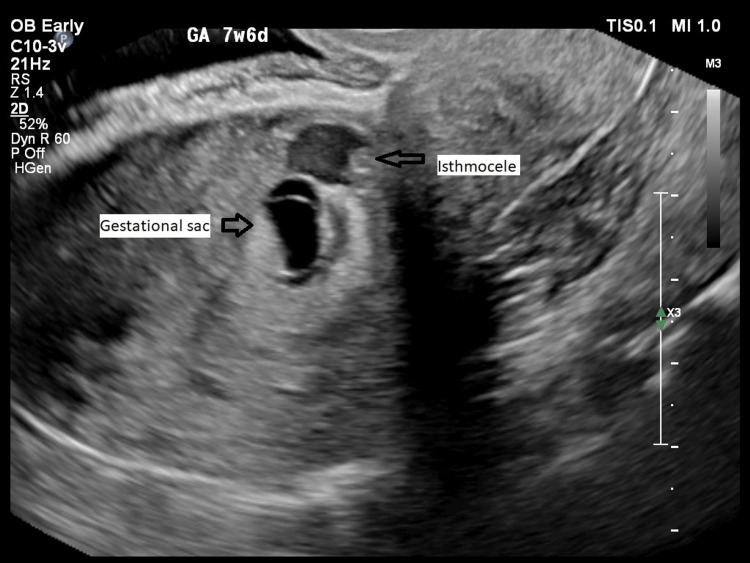
CSEP embedded in the myometrial scar defect (isthmocele) CSEP: Cesarean scar ectopic pregnancy An original sample image from the study.

A total of 633 patients underwent dating scans over a one-year period, resulting in six cases of cesarean scar ectopic pregnancy (CSEP), yielding a prevalence of 0.9%. The mean age of the patients was 32.8 ± 3.2 years, and the mean gestational age at diagnosis was 6.3 ± 0.5 weeks. All CSEP cases were diagnosed by transvaginal ultrasound (Figure 1).

**Figure 2:**
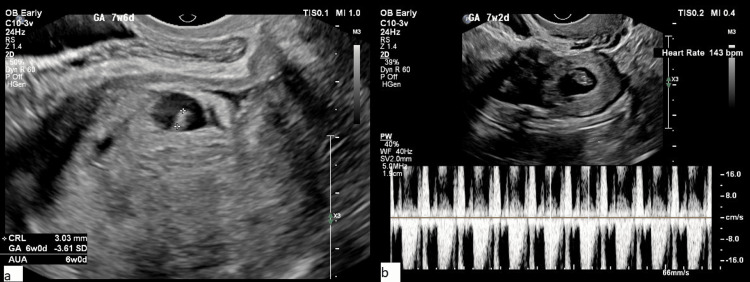
Sample images showing fetal pole and cardiac activity (a) Cesarean scar ectopic pregnancy (CSEP) with the presence of fetal pole and (b) cardiac activity. Original images from the study.

Four out of six patients had a previous history of more than one cesarean section (66.7%). Four patients had a viable CSEP with the presence of fetal pole and cardiac activity (Figure 2).

**Figure 3:**
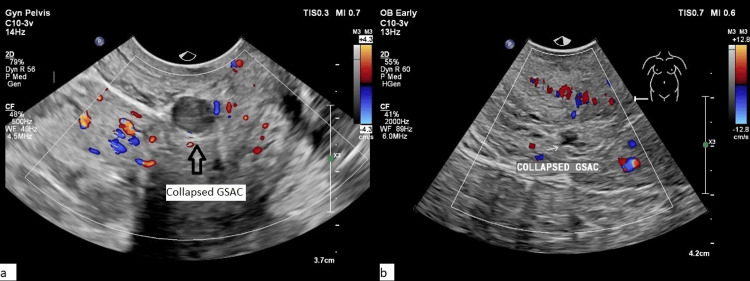
Resolution of the gestational sac in two cases (a) and (b) Resolution of the gestational sac (Gsac) following intracardiac instillation of KCl and intrasac methotrexate instillation, demonstrated in two different cases. Original images from the study.

All cases of viable CSEP underwent intracardiac injection of potassium chloride (KCl) followed by methotrexate instillation into the gestational sac. Three of these injections were administered transvaginally, while one was performed transabdominally. This approach successfully terminated fetal cardiac activity and led to the collapse of the gestational sac (Figure 3).

Two patients underwent curettage under ultrasound guidance where internal os was open, and no cardiac activity was documented.

Intracardiac injection of KCL was the modality of choice for cases with fetal cardiac activity. Curettage was performed when fetal cardiac activity was absent, the internal os was open, and the ectopic sac did not extend beyond the serosal layer of the uterus. Serum human chorionic gonadotropin (hCG) levels were monitored on days one, four, and seven. A decrease of 15% between days four and seven was considered satisfactory. If the decrease was less than 15%, an additional dose of intramuscular methotrexate (1 mg/kg) was administered. All patients recovered successfully without requiring major surgery. The findings of our study are listed below (Table 1).

**Table 1:** Management strategies and outcomes of the patients in our study KCl: Potassium chloride, MTX: Methotrexate, BHCG: Beta-human chorionic gonadotropin.

Characteristics	Case 1	Case 2	Case 3	Case 4	Case 5	Case 6
Age	39	30	33	34	29	32
Number of previous sections	2	1	2	2	2	1
Gestational age	6 weeks 2 days	7 weeks	7 weeks	6 weeks 5 days	6 weeks	5 weeks 6 days
Presence of cardiac activity	No fetal pole	Fetal pole with the presence of cardiac activity	Fetal pole with the presence of cardiac activity	Fetal pole with the presence of cardiac activity	Fetal pole with no cardiac activity	Fetal pole with the presence of cardiac activity
Primary treatment	Ultrasound-guided curettage	Intra cardiac instillation of KCl and intrasac MTX	Intra cardiac instillation of KCl and intrasac MTX	Intra cardiac instillation of KCl and intrasac MTX	Ultrasound-guided curettage	Intra cardiac instillation of KCl and intrasac MTX
Secondary treatment	no	no	no	Yes, curettage	Yes, repeat curettage	no
Time to reach negative BHCG	5 weeks	6 weeks	6 weeks	6 weeks	5 weeks	5 weeks

## Discussion

The clinical presentation of CSEP may vary from asymptomatic patients with positive urine pregnancy tests to acute presentations such as pelvic pain, bleeding per vaginum, uterine rupture, and hemodynamic instability. Transvaginal ultrasound has a sensitivity of 85% for the detection of CSEP [6].

Various treatment options have been described in the literature. However, the rarity of the condition and paucity of literature limits any definitive treatment recommendation. A literature review on CSEP describes five management approaches: expectant management, medical treatment, surgical treatment, uterine artery embolization, or a combination approach. Expectant management is associated with morbid complications such as hemorrhage, uterine rupture, preterm delivery, etc. [7]. The Society of Fetal Medicine and the American College of Obstetricians and Gynecologists do not recommend expectant management for definitive CSEP diagnosis [8]. 

Medical treatment

This encompasses intragestational injection of methotrexate with or without accompanying systemic methotrexate. Jurkovic et al. reported 18 cases of CSEP over four years duration, where success rates of medical treatment were 71% (five out of seven patients), surgical treatment was 100%, and expectant management was 33%. Out of the seven patients undergoing medical treatment, five patients received local methotrexate, one patient received local KCl, and one patient received a combination of both. Two patients in whom medical treatment was unsuccessful received local methotrexate only [9].

In our study, we administered a combination of local KCl and methotrexate (KCl into the fetal heart and methotrexate into the gestational sac) in four patients. All these patients had fetuses with cardiac activity. This medical treatment led to the successful resolution of cardiac activity and the collapse of the gestational sac (Table 1). One patient required a curettage in view of persistent mild bleeding per vaginum. We did not administer systemic methotrexate to any of the patients.

The Society of Fetal Medicine and American College of Obstetricians and Gynecologists recommend intragestational methotrexate for medical treatment of CSEP, with or without other treatment modalities (GRADE 2C). Systemic methotrexate alone should not be used to treat CSEP [8]. The resolution of the gestational sac may take several weeks. There may also be a transient increase in hCG levels and CSEP mass size. The patient should be monitored for alarming symptoms like severe pain in the abdomen, heavy bleeding per vaginum, loss of consciousness, etc. A transvaginal ultrasound may be performed after six to eight weeks to document the resolution of the gestational sac and cardiac activity.

The technique of intracardiac injection

Under ultrasound guidance and using a transvaginal approach, a 20-gauge needle is inserted into the gestational sac and then into the fetal heart. Injection KCl (2 ml) is injected into the fetal heart. Fluid is aspirated from the gestational sac, and Injection methotrexate in the dose of 1 mg/kg of maternal weight is instilled into the gestational sac. 

**Video 1:** USG guided needle insertion into the fetal heart for instillation of injection KCl USG: Ultrasonography, KCl: Potassium chloride Original procedure performed in the study.

Surgical treatment

Various surgical treatment modalities are available for CSEP, including cesarean scar resection, laparoscopy, laparotomy, and curettage. Cesarean scar resection can be performed either via transvaginal route or by laparoscopy. During this procedure, the scar tissue is excised, and the surrounding myometrium is reapproximated. This approach is associated with lower complication rates compared to other surgical options, although the available data are variable [8,10]. He et al. reported six successful cases of transvaginal removal of ectopic pregnancy tissue, followed by repair of the uterine defect [11].

Ultrasound-guided suction curettage is another surgical option for CSEP. However, it carries risks such as uterine perforation, especially in exogenic-type CSEP, and excessive bleeding due to abnormal placentation. In a retrospective study on 232 women diagnosed with CSEP, 82.3% of women were treated by ultrasound-guided suction curettage; 4.7% of women required blood transfusion; one patient required hysterectomy due to uncontrollable intraoperative bleeding, and 6.0% had retained products of conception and required repeat curettage. A modified Shirodkar suture was used to achieve hemostasis if required [12].

Owing to the risks of hemorrhage, we opted for ultrasound-guided suction curettage only in cases where (1) internal os was open, (2) the fetus was non-viable, (3) endogenic type CSEP, and (4) persistent bleeding per vaginum. In our study, two of the six patients underwent ultrasound-guided curettage and one of them required a repeat curettage due to persistent bleeding (Table 1). The Society of Fetal Medicine recommends operative resection (transvaginal) or ultrasound-guided uterine aspiration for the surgical management of CSEP, with sharp curettage alone being avoided (GRADE 2C) [8].

Uterine artery embolization (UAE)

**Figure 4:**
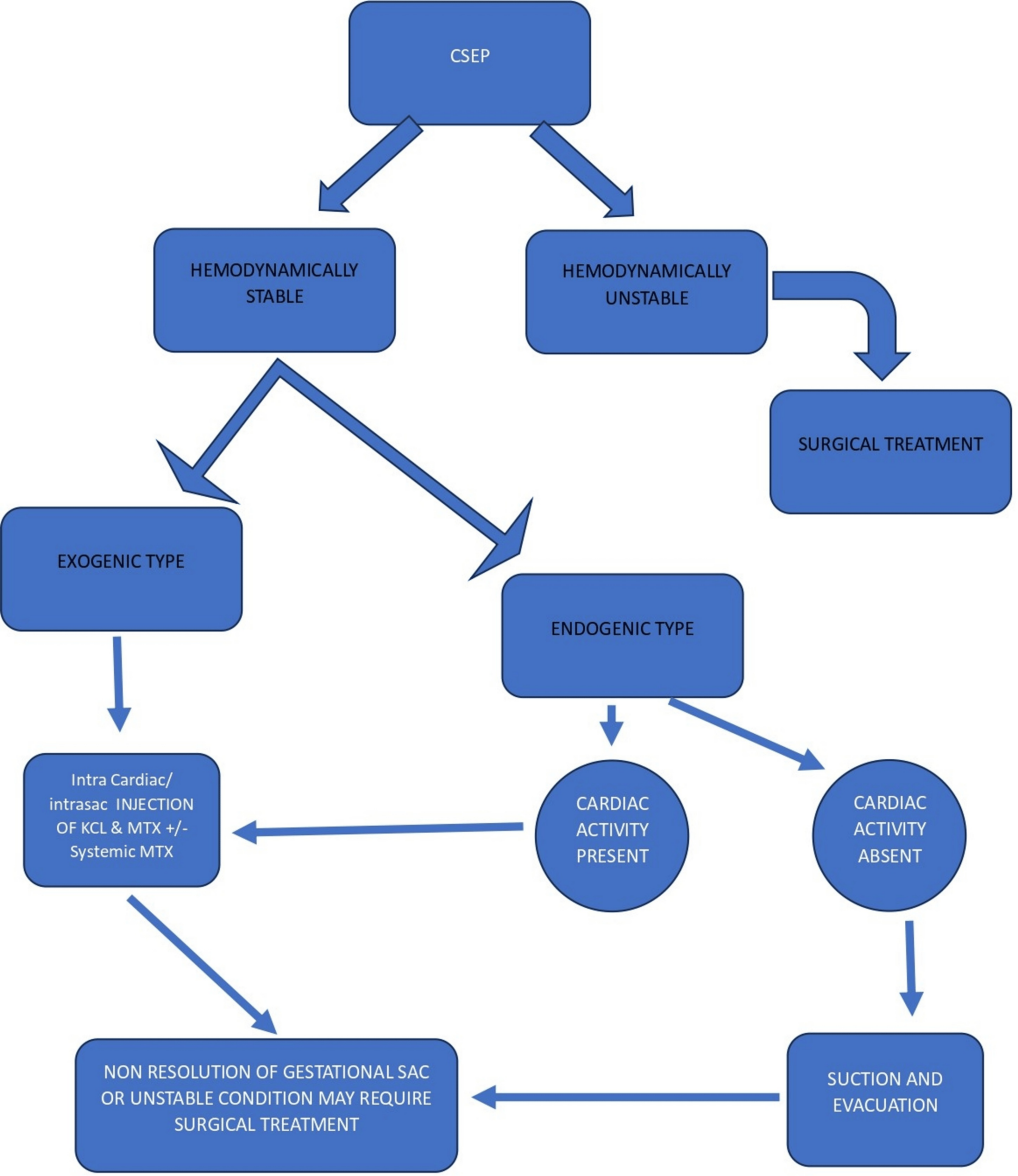
Treatment approach and management of cesarean scar ectopic pregnancy KCl: Potassium chloride, MTX: Methotrexate. Original treatment flowchart based on the study.

Uterine artery embolization (UAE) is a minimally invasive technique used to manage obstetric and gynecological hemorrhage, including postpartum hemorrhage (PPH), uterine fibroids, and ectopic pregnancies. Several studies have reported the successful use of UAE in combination with other modalities, such as methotrexate and suction curettage, for the treatment of cesarean scar ectopic pregnancy [13-15]. UAE can help reduce the risk of hemorrhage in patients who subsequently undergo curettage. A case report has described the use of UAE followed by hysteroscopic diode laser resection as a successful treatment modality for CSEP [16]. A flowchart illustrating the treatment approach for CSEP based on our study is provided below (Figure 4).

Study limitations

The limitations of this study include a small sample population and its conduct in a single tertiary center. Because all patients underwent medical management, we were unable to compare our outcomes with surgical treatment options.

## Conclusions

Cesarean scar ectopic pregnancy (CSEP) presents a diagnostic and therapeutic challenge. A high index of suspicion is crucial, particularly in patients with a history of cesarean delivery, even in the absence of symptoms. While high-quality transvaginal ultrasound aids in diagnosis, CSEP can often be overlooked.

In our experience, intrasac/intracardiac instillation of potassium chloride (KCl) and methotrexate (MTX) is a convenient and effective management option for CSEP. This approach is preferred due to its safety, ease of performance, avoidance of anesthesia and major surgery, and potential for improved obstetric outcomes in future pregnancies. In hemodynamically stable patients, this medical treatment modality can be adopted as the first line of management. However, surgical modalities remain the preferred choice for hemodynamically unstable patients or those seeking definitive treatment for CSEP.
